# Yellow Fever—Clinical Notes

**Published:** 1899-06

**Authors:** 


Yellow Fever—Clinical Notes, by Just Touatre, M. D. (Paris), formerly Physician-in-chief to the French Society Hospital, New Orleans; member Board of Experts Louisiana Board of Health. Translated from the French by Chas. Chassaignac, M. D., editor *New Orleans Medical and Surgical Journal,* etc. Published by New Orleans Medical and Surgical Journal Company, limited. Pages, 200. Price, ——.

This little work is a parting gift from Dr. Touatre upon his return to his native land after having spent the greater part of a useful and active professional life at New Orleans. While there, and having charge of the French Hospital and acting as expert for the Louisiana State Board of Health, he probably had as much experience in the treatment of yellow fever as any one now living, and that he has given his readers the full benefit of his experience it only needs a perusal of his book to demonstrate. The work is altogether clinical in its scope. The writer has never seen a case of yellow fever, but it seems to us that it would almost be impossible to fail to recognize one after reading Dr. Touatre's description. J.

